# Adipose Tissue Metabolism and Cancer Progression: Novel Insights from Gut Microbiota?

**DOI:** 10.1007/s40139-017-0154-6

**Published:** 2017-10-24

**Authors:** Benedicte F. Jordan, Florian Gourgue, Patrice D. Cani

**Affiliations:** 1 Louvain Drug Research Institute, Biomedical Magnetic Resonance Research group, Université Catholique de Louvain, Av. E. Mounier, 73, B1.73.08, 1200 Brussels, Belgium; 2 Louvain Drug Research Institute, WELBIO (Walloon Excellence in Life Sciences and BIOtechnology), Metabolism and Nutrition Research group, Université Catholique de Louvain, Av. E. Mounier, 73 box B1.73.11, 1200 Brussels, Belgium

**Keywords:** Gut microbiota, Cancer progression, Adipose tissue, Adipokines, Inflammation, Bacteria

## Abstract

**Purpose of Review:**

Obesity is strongly associated with the development of several types of cancers. This review aims to discuss the recent key mechanisms and actors underlying the link between adipose tissue metabolism and cancer, and the unequivocal common mechanisms connecting gut microbes to adipose tissue and eventually cancer development.

**Recent Findings:**

Complex interactions among systemic and tissue-specific pathways are suggested to link obesity and cancer, involving endocrine hormones, adipokines, fatty acids, inflammation, metabolic alterations, and hypoxia. Emerging evidence also suggests that the gut microbiota, another key environmental factor, may be considered as a converging element. Studies have shown that cancer susceptibility may be induced in germ-free mice colonized with the gut microbiota from high-fat diet-fed mice. Suggested mechanisms may involve inflammation, immunity changes, lipogenic substrates, and adipogenesis.

**Summary:**

Cancer development is a complex process that may be under the control of previously unthought factors such as the gut microbiota. Whether specific intervention targeting the gut microbiota may reduce adipose tissue-driven cancer is an interesting strategy that remains to be proven.

## Introduction

Obesity is associated with diabetes and cardiometabolic disorders. The global burden disease (GBD) study recently reported that obesity is undoubtedly epidemic and as now reached more than 600 million people [1]. Obesity is characterized by a massive adipose tissue expansion together with specific changes at the level of immunity, the presence of a low-grade inflammatory tone (i.e., macrophage infiltration and cytokines production), and an altered fatty acid storage. Besides the development of metabolic disorders such as diabetes and cardiovascular diseases, obesity is strongly associated with the development of several types of cancers [2••].

Nowadays, it is commonly accepted that different modifiable risk factors such as diet and sedentary life style, alcohol and tobacco play a major role in the pathogenesis of cancer. Strikingly, emerging evidence suggests that another key environmental factor may be considered as a converging element: that is the gut microbiota.

Gut microbes have gained remarkable attention over the last 10 years. Metagenomic (i.e., sequencing of the overall genome of gut bacteria) and metabolomic (e.g., comprehensive analysis of metabolites and specific molecule) approaches have helped to elucidate the composition and the metabolic activity of the gut microbiota. It is estimated that trillions of microbes inhabit the human gut, which represent at least a 1:1 ratio between human and microbial cells, or even more [3]. Today, a vast number of publications have associated the composition and the metabolic activities of the microbiota with many diseases and organs (e.g., brain, liver, fat, gut, muscles) [4]. It is indeed established that gut bacteria participate not only to shape our immunity but also control the regulation of glucose, energy, and adipose tissue metabolism via several mechanisms including inflammation [5•, 6•]. It is important to note that the metabolic features involving the development of adipose tissue during obesity are for most of them also associated with the development of cancer. However, the exact mechanisms explaining the obesity-cancer link are not elucidated.

This review aims to discuss (1) the recent key mechanisms and actors underlying the link between adipose tissue metabolism and cancer, and (2) the unequivocal common mechanisms connecting gut microbes to adipose tissue metabolism and eventually cancer development.

## Adipose Tissue Metabolism and Fat Storage

**Fig. 1:**
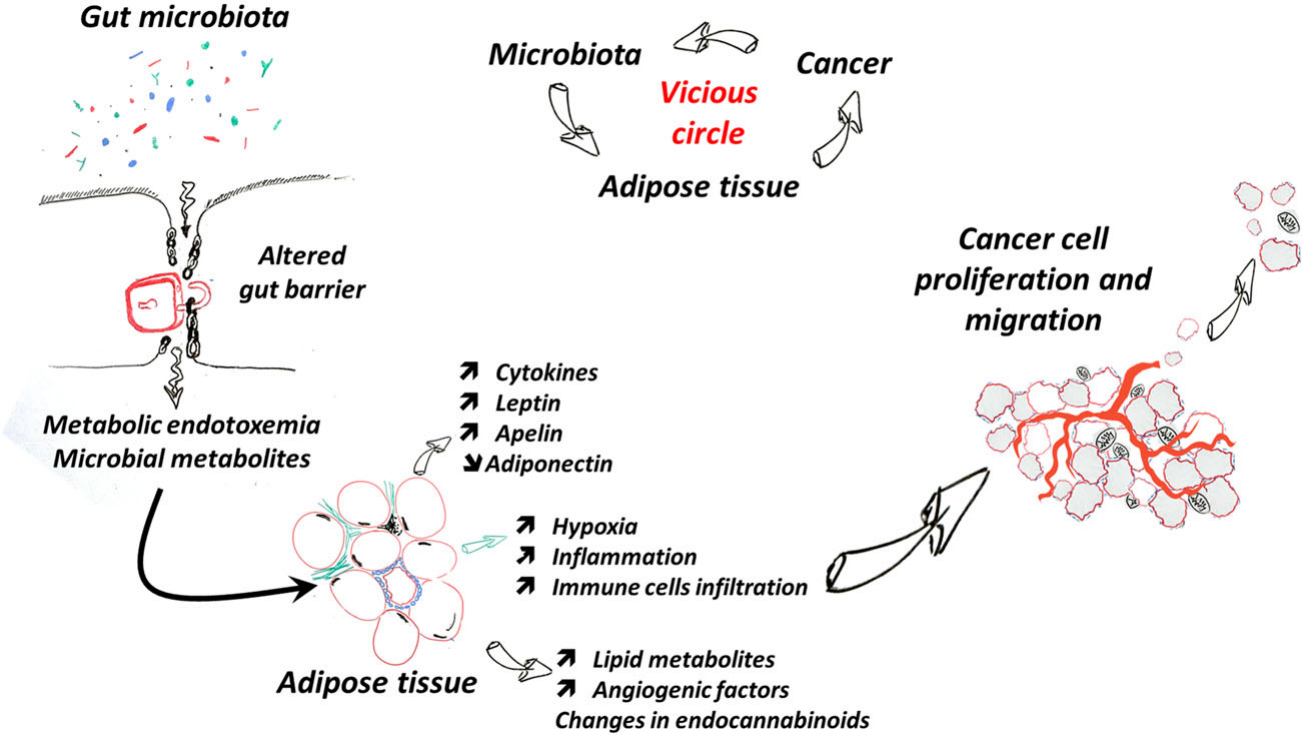
Hypothetical working models of interactions between adipose tissue metabolism, gut microbiota, and cancer progression. Adipose tissue and cancer development are sharing several similar mechanisms and stimulatory factors. The adipose tissue metabolism is profoundly affected during obesity and metabolic disorders, where the different cells composing this tissue are secreting cytokines, adipokines, and lipid mediators that are triggering key metabolic processes devoted not only to reverse hypoxia but also to expand the adipose tissue for further fatty acid storage. Among the key actors, the so-called metabolic endotoxemia which is triggered by the disruption of the gut barrier and gut microbiota alteration is known to trigger low-grade inflammation, to increase apelin and leptin secretion, and to change the endocannabinoid system. All these changes are key elements contributing to a cascade of events that also profit to cancer cells, thereby leading to an appropriate environment stimulating cell proliferation, angiogenesis, and eventually cell migration and metastatization. In turn, data also suggest that cancer development contribute to change the gut microbiota and support the formation of a vicious circle

Adipose tissue is a complex organ which is composed of different cell types. More precisely, preadipocytes, vascular endothelial cells, stroma cells, pericytes, and infiltrating blood cells (i.e., monocytes, macrophages, lymphocytes, and mast cells) are in perpetual interaction to fine tune tissue expansion and metabolic responses [7, 8]. In fact, adipose tissue is constantly subjected to the slight remodeling of its extracellular matrix. However, during a prolonged positive energy balance, numerous changes will occur such as new adipocyte formation, adipocyte hypertrophy, angiogenesis processes (neovascularization), infiltration of immune cells, and an overall process considered as “physiological” adipose tissue expansion [7, 8]. The adjustment of such metabolic controls is essential for the maintenance of adequate energy storage and adipose tissue expansion. Nevertheless, chronic energy overload instead of adapting to the demand of the expanding tissue may lead to inadequate remodeling of the extracellular matrix. In parallel, the vasculature is critical for the development of the adipose tissue and the storage of energy [9]. Importantly, insufficient angiogenesis blocks adipose tissue accumulation, thereby resulting in relative hypoxia and contributes to the production of many factors from both adipocytes and other cells composing the adipose tissue [10] (Fig. 1). Data also show that low-grade inflammation leads to chronic impairment of adipogenesis processes, activation of myofibroblasts, macrophages, thereby promoting the accumulation of abnormal extracellular matrix, tissue hypoxia, and the production of angiogenic factors [11–14]. However, chronic inflammation might also partially be a response due to hypoxia since inflammatory cytokines, macrophage migration inhibition factor (MIF), TGF-β, and matrix metallopeptidase 9 (MMP9) were induced by hypoxia in primary adipocytes and macrophages prepared from adipose tissue of lean mice [15]. White adipose tissue in transgenic and dietary obese mice indeed show reduction in the interstitial partial oxygen pressure, and similar observations were made between adipose tissue of obese and lean humans [16].

## Fat Cells and Cancer

Besides the adipose tissue, other adipocyte-rich organs are suggested to create microenvironments that are conducive for tumorigenesis and metastatic progression [17]. Numerous epidemiological data have indeed shown that obesity is associated with increased incidence of several cancers and more precisely, breast, endometrium, ovarian, colon, gastro-intestinal, renal, prostate, thyroid, and hematological malignancies [18–20, 21••]. In countries where obesity prevalence has increased rapidly, a significant proportion (~ 20%) of all new cancers may be attributable to obesity [22]. Obesity in children from 2 to 14 years is also associated with increased cancer risk in adulthood by 40% [18]. In addition, weight control interventions have been shown to be able to reduce cancer incidence in women [19]. Therefore, obesity is now projected to replace tobacco as the most common modifiable risk factor for the development of malignancies [21••]. Interestingly, peri-tumoral fat has been shown to correlate with lymph node invasion in breast cancer and in esophageal adenocarcinoma [23]. The mechanisms linking increased adiposity to malignancy are not completely understood. Complex interactions among systemic and tissue-specific pathways are suggested and involve endocrine hormones, adipokines, fatty acids, inflammation, metabolic alterations (i.e., hyperglycemia and hyperinsulinemia), hypoxia and oxidative stress [24••, 21••], and potentially, the gut microbiota [19, 25••]; the later will be further discussed in the last part of this review. In the next part of this review, we will describe how the adipose tissue and its specific product may directly contribute to cancer development.

## The Adipose Tissue Microenvironment Favors Tumor Initiation and Progression

Adipocytes indeed represent a significant source of lipids, cytokines, and adipokines, and their presence in the tumor microenvironment can affect cell signaling and metabolism [17]. The next paragraphs summarize the recent findings linking adipokines and adipose tissue metabolism with cancer development (Fig. 1).

### Adipokines

Undeniably, adipokines, such as *leptin* or resistin, promote cancer cell progression via enhancement of cell proliferation and migration, inflammation, promitogenic, and anti-apoptosis pathways, which subsequently can prompt tumor growth and metastatization [26]. Cell proliferation and angiogenic pathways that are affected by adipokines involve JAK/STAT, MAPK/ERK, PI3K/mTOR, cyclins, and VEGF [21••, 22]. Leptin has recently been shown to play a role in metabolic reprogramming in breast cancer cells, consisting of an enhanced use of glucose for biosynthesis and lipids for energy production, as a result of the increased demands of energy and biosynthetic intermediates to sustain proliferation and invasion [27]. Other potential metabolic adaptations induced by leptin include stabilization of HIF1-alpha in hypoxic conditions, increased uncoupled respiration, and elevated expression of MCT4 lactate exporter [28]. Besides adipocytes, cancer-associated fibroblasts (CAFs), the principal cellular component of the stroma, also express leptin receptor and secrete leptin, which sustains a short autocrine loop and is able to target tumor epithelial cells enhancing cancer cell growth and invasiveness. Recent results show that activated farnesoid X receptor (FXR) may be able to counteract the leptin-dependent paracrine effects on breast cancer restraining the tumor-promoting activities exerted by CAFs [29]. Serum leptin levels have been shown to correlate with the incidence of some cancers (breast, endometrial, colorectal, prostate), while other studies showed a lack or an inverse relation with cancer risk [21••, 30]. Increased expression of the leptin receptor (ObR) has been correlated with decreased survival in ovarian cancer and the development of distant metastases in breast cancer [21••]. Finally, leptin also induces overexpression of leptin, Ob-R, estrogen receptor, and aromatase mRNA, suggesting the possible involvement of leptin in estrogen pathway [31].

*Apelin* is another adipokine whose levels are increased not only with obesity [32] but also in several cancers [33, 34]. This adipokine acts through the activation of its receptor (APJ) to enhance tumor angiogenesis [35]. In addition, apelin can bind to APJ expressed on lymphatic endothelial cells to increase tumor lymphangiogenesis and metastatization [36]. Furthermore, apelin has been suggested as a prognostic marker for cancer progression as its levels correlate with cancer invasion [34].

Obesity is also commonly associated with decreased *adiponectin* levels in the circulation, for which an inverse relationship with the risk of development and progression of multiple cancers has been described [21••, 37]. Adiponectin can indeed inhibit cell proliferation, induce apoptosis, and decrease invasion of tumor cells through the activation of multiple signaling pathways downstream of the adiponectin receptors, AdipoR1 and AdipoR2, including AMPK, PI3K/mTOR, and the nuclear factor-kappaB (NF-kB). In addition, adiponectin has also recently been shown to inhibit CREB (cyclic AMP response element-binding transcription factor) activation in lung adenocarcinoma cells [38]. This effect further highlights the anti-tumoral potential of adiponectin as high levels of CREB have been identified in prostate cancer, breast cancer, non-small-cell lung cancer (NSCLC), and leukemia and correlates with cancer cells differentiation and poor prognosis [39].

Recently, it has been discovered that the circulating levels of *survivin*, a member of the inhibitor of apoptosis (IAP), are increased in obese patients [40].

Survivin was already known to be increased in many cancers [41] and to have a broad pro-tumoral activity by its association with multiple cell signaling pathways including PI3K/mTOR, ERK, MAPK, STAT, or HIF-1α. Hence, survivin correlates with tumor invasion and metastatization, enhances VEGF expression to promote tumor angiogenesis, and interferes with chemo- and radiotherapy by inhibiting apoptosis [42].

### Lipid Metabolism

Alterations in the lipid metabolism are part of the reprogrammed energy metabolism that characterizes cancers [43]. Indeed, lipids are essential component for tumors to generate cellular membranes, lipid-derived bioactive molecules and also to produce energy through mitochondrial fatty acids oxidation. In many cancers cells, upregulation or increased activity has been described for key enzymes and transporters of the lipid pathway [43, 44•]. For example, fatty acid-binding protein 4 (FABP4) is a key adipokine for fatty acid transport and plays a role in tumor progression of breast cancer among others by enhancing proliferation of breast cancer cells, with no impact on cell migration, likely via activation of the AKT and MAPK signaling cascades [45]. FABP9, another member of the FABP family, is increased in prostate cancer patients and correlates with reduced survival [46]. The fatty acid receptor CD36 is also unique in its ability to initiate metastasis. Clinically, the presence of CD36+ metastasis-initiating cells correlates with a poor prognosis for numerous types of carcinomas, and inhibition of CD36 also impairs metastasis, at least in human melanoma- and breast cancer-derived tumors [47].

Beside fatty acids uptake, it has been shown that many cancer cells upregulates acetyl-Coa carboxylase (ACC) and fatty acid synthase (FASN) expression to increase de novo lipogenesis and that these high levels correlate with poor prognosis [44•]. One particularity of cancer cells regarding the lipid metabolism is their ability to signal to adipocytes from their microenvironment to induce a modification of adipokines secretion as well as a delipidation of these so-called cancer-associated-adipocytes (CAA). The released fatty acids can be used by cancer cells for β-oxidation or as metabolic substrates to sustain a highly proliferative state and support cancer cells migration [48].

### Low-Grade Inflammation

Hypertrophic adipose tissue from obese patient is known to recruit macrophages and to have an unbalanced ratio between M1 pro-inflammatorymacrophages and M2 anti-inflammatory macrophages. The growing M1 population secrete inflammatory cytokines including TNF-α and IL-6 leading to a chronic low-grade inflammation that promote ROS production and therefore genetic instability, cancer cells proliferation, tumor angiogenesis, and metastatization [49].

Similar to the adipose tissue, tumors also include macrophages called tumor-associated-macrophage (TAM). The role of TAMs in cancer progression has been intensively studied and reviewed elsewhere [50••]. Briefly, TAMs support tumor progression by growth factors and ROS production, increased angiogenesis and lymphangiogenesis, and tissue infiltration and by creating an immunosuppressive environment.

## Gut Microbes Regulate Adipose Tissue Metabolism: A Link with Cancer?

All the processes regulating adipose tissue metabolism and described in the first part of this review have also been linked with gut microbiota activity. For example, seminal papers from Backhed and colleagues have shown that gut bacteria allow energy harvesting from nutrients ingested but not digested by the host [51]. They found that germ-free mice (i.e., mice raised in the absence of microorganisms) where leaner, with about 40% less total body fat than mice with a normal gut microbiota. Strikingly, this is observed even if the germ-free mice ate 30% more diet than mice harboring a gut microbiota. The mechanisms of this obvious weight gain due to microbes are not only the increase in energy extraction from non-digestible food component but also a higher intestinal glucose absorption and concomitant higher glycemia and insulinemia, which are two major metabolic factors regulating lipogenesis and fat storage. In addition, glucose and insulin promote de novo lipogenesis through the expression of several key enzymes such as ACC and FAS. This is also associated with adipocyte hypertrophy mainly through an increase fatty acid synthesis and storage depending on the activity of the enzyme lipoprotein lipase (LPL) which catalyzes the release of fatty acids from circulating lipoproteins [51]. Finally, transferring the gut microbiota from obese mice into germ-free mice reproduces the phenotype of fat mass accumulation and altered adipose tissue metabolism, strongly suggesting a direct link between the presence of specific microbes in the gut and the development of adipose tissue [52, 53].

On top of these physiological processes, numerous evidence suggests that gut microbes contribute to the onset of low-grade inflammation and an altered regulation of lipogenesis and adipogenesis processes (i.e., angiogenesis, differentiation, fat expansion), thereby leading to larger adipocytes, abnormal fat accumulation, and hypoxia (Fig. 1) [54•].

In 2007, the concept of metabolic endotoxemia was discovered. It was shown that the low-grade inflammatory tone characterizing obesity, diabetes, and related metabolic disorders is likely due to the translocation of specific proinflammatory molecules from the gut microbiota into the bloodstream. This factor is the so-called lipopolysaccharide (LPS) which initiates inflammation, insulin resistance, and metabolic disorders [55]. Thus, metabolic endotoxemia is defined as a modest increase in circulating LPS, high enough to be involved in the onset of diseases (Fig. 1). This key mechanism explains how gut microbes and eventually gut barrier function interfere with numerous factors regulating fat mass development.

Among the metabolic systems involved in the regulation of this gut barrier and adipogenesis, it has been shown that endocannabinoids (eCBs) plays a major role (for review, [54•]). This system is composed of several bioactive lipids belonging to the *N*-acylethanolamines and acylglycerol families. Importantly, specific eCBs have been shown to control not only adipogenesis and inflammation but also angiogenesis processes.

Interestingly, the beneficial effects of specific eCBs on adipose tissue metabolism are counteracted by LPS, thereby leading to adipocyte cell hypertrophy and secretion of adipokines [11, 56]. It has also been demonstrated that the production of apelin by the adipose tissue is directly regulated by both LPS coming from the gut and eCBs [56]. More specifically, LPS completely abrogated the physiological downregulation of apelin and its receptor APJ induced by eCBs. Therefore, it is suggested that both eCBs and LPS are implicated in adipose tissue metabolism.

Moreover, alteration of specific eCBs may also drive the development of tumors and metastasis [57•]. Therefore, data suggest that the gut microbiota and metabolic endotoxemia strongly contribute to the regulation of eCBs tone and eventually may interfere with cancer development. Data suggest that leptin sensitivity and levels may be controlled by the gut microbiota, similar to apelin. As an example, it has been shown that high-fat diet feeding changes the microbiota composition and is associated with a higher leptin production. Everard et al. have shown that changing the gut microbiota by using prebiotics not only changes the gut microbiota composition, reduces LPS, reduces leptin levels and increases leptin sensitivity [58]. Along this line, germ-free mice are also more sensitive to leptin, whereas the colonization of their intestine with gut bacteria increases leptin production and leptin resistance [59]. Thus, as discussed above, the gut microbiota is known to affect adipose tissue metabolism, cellular proliferation, inflammation, and immunity. Consequently, it is also suggested to regulate cancer at the level of predisposing conditions such as initiation and progression. Definitive evidence for the role of particular species in cancer pathogenesis would require more studies [60••]. Nevertheless, preclinical data suggest a potential link between microbiota, obesity, and cancer [58]. For example, several studies have shown that cancer susceptibility may be induced in germ-free mice colonized with the gut microbiota from high-fat diet-fed female mice, and this even if the recipient mice were fed with a normal diet [61•]. More strikingly, this effect was also observed in an intergenerational manner. Thus, this observation strongly suggests that that factors associated with adipose tissue metabolism and the gut microbiota contribute to the onset of cancer (Fig. 1).

Recently, it also become evident that the gut microbiota modulates the response to cancer therapy and susceptibility to toxic side effects [25••]. Within the scope, the transfer of the fecal microbiota of patients who are responsive to cancer therapy into germ-free mice has been shown to endow those animals with an ability to respond efficiently to the therapy [62]. Therefore, it can be speculated that host genetics and lifestyle in part may indirectly affect carcinogenesis and response to cancer therapy through modification of microbiota composition [25••].

## Conclusions

Strong evidence exist regarding the impact of microbiota on metabolic activities and specific cancer such as colon cancer. This is indeed easy to understand because of the close vicinity of microbial cells and their metabolites with colonic cells. However, it is less evident to directly link the development of cancers starting at distance from the gut with the gut microbiota. Nevertheless, evidence suggests that processes of adipose tissue expansion characterizing obesity are also influenced by the gut microbiota, mainly through inflammatory mechanisms, immunity changes, lipogenic substrates, and adipogenesis (Fig. 1). Hence, the adipose tissue and its metabolic activity during obesity (i.e., adipokines secretion, angiogenesis, hypoxia) may directly influence cancer progression. More impressively, as discussed in this review not only inflammation but also numerous adipokines produced by the adipose tissue are linked to cancer initiation, progression and processes of metastatization. Therefore, it is important to consider that cancer development is a complex process that may be under the control of previously unthought factors such as the gut microbiota. Whether specific intervention targeting the gut microbiota may reduce adipose tissue-driven cancer is an interesting strategy that remains to be proven.
